# The Effect of a Modified Tenodesis Wrist-hand Orthosis on Hand Function in Patients With Tetraplegia

**DOI:** 10.33137/cpoj.v7i1.42879

**Published:** 2024-10-28

**Authors:** S.P Sonune, A Saha, N.G Joshi, S Pathak, P Bhadra, G Goel

**Affiliations:** Department of Physical Medicine and Rehabilitation, All India Institute of Medical Sciences, Bhopal, India.

**Keywords:** Hand Orthosis, Wrist Hand Orthosis, Spinal Cord Injury, Tetraplegia, Hand Rehabilitation, Orthosis

## Abstract

**BACKGROUND:**

An individual experiencing tetraplegia faces functional limitations due to impaired hand function. The use of an affordable tenodesis wrist-hand orthosis (WHO) can enable finger flexion with active wrist extension, thereby enhancing the three-jaw chuck grasp and overall hand functionality.

**OBJECTIVES:**

To assess hand function and satisfaction in patients with tetraplegia using a modified tenodesis wrist-hand orthosis (WHO), utilizing the Duruöz Hand Index (DHI) and the Orthotics and Prosthetics User Survey (OPUS) satisfaction with device and services subscales.

**METHODOLOGY:**

The study was conducted at a tertiary care center in central India, enrolling patients with tetraplegia admitted to the Department of Physical Medicine and Rehabilitation. A modified tenodesis wrist-hand orthosis (WHO) was designed using low-temperature thermoplastic components. Twenty-two individuals with a minimum wrist extensor power of grade 3/5 were included in the study. These patients were provided with the modified tenodesis WHO and underwent daily training sessions for a period of 2 weeks. Duruöz Hand Index (DHI) scores were assessed at baseline, 6 weeks, and 12 weeks postenrolment. Patient satisfaction was evaluated using the Orthotics and Prosthetics User’s Survey (OPUS) satisfaction with device and services subscales.

**FINDINGS:**

The analysis of the DHI scores indicated a significant enhancement in functional abilities at both 6-week and 12-week follow-ups compared to the baseline assessment. Notably, the most substantial progress at 6 weeks follow-up was observed in tasks such as buttoning a shirt, while significant improvement at the 12-week mark was noted in activities like turning a key in a lock. The median OPUS device satisfaction score was 50, corresponding to a Rasch score of 68.8. Additionally, the median OPUS satisfaction score for services stood at 46, with a Rasch score of 72.7. Patients expressed the highest satisfaction levels with the courteous demeanor of the staff, prompt scheduling of appointments, and accurate fitting of the orthosis.

**CONCLUSION:**

The study findings indicate that the modified tenodesis WHO is an effective and satisfactory therapeutic device for improving hand function in patients with tetraplegia. The findings encourage further investigation and application of the modified tenodesis WHO in clinical practice.

## INTRODUCTION

Spinal cord injury (SCI) is a complicated and incapacitating disorder that often leads to significant functional loss. India reports approximately 20,000 new cases of spinal cord injury (SCI) each year, with a majority affecting young individuals from lower socioeconomic backgrounds.^1^

The prevalence of traumatic cervical spine fractures in Western populations ranges from 4 to 17 cases per 100,000 person-years, with concurrent cervical spinal cord injuries (SCI) occurring in 10–11% of cases.^2^ Loss of hand function is one of the most notable areas of impairment for people with cervical SCI, and it significantly affects their everyday activities and quality of life. The upper limb function, particularly hand grasp and release, is critical for performing essential tasks of daily living, social interaction, and vocational activities. Consequently, restoring hand function is a primary objective following cervical spinal cord injury (SCI). A study by Anderson involving 347 individuals with cervical-level SCI found that 48.7% of patients prioritized regaining arm and hand function as the most impactful improvement for their quality of life, ranking it above sexual function, trunk stability, bowel and bladder control, and walking.^3^ Despite advancements in medical management and rehabilitation, many individuals with SCI still face significant challenges in regaining optimal hand function.

Tenodesis grasp and release is a biomechanical phenomenon that occurs with wrist extension and flexion and plays a crucial role in enabling functional grasp and release movements in individuals with SCI. The tenodesis effect is used in patients with tetraplegia with innervated wrist extensor muscles but paralyzed finger and thumb flexor muscles. When the wrist is actively extended, passive tension of the extrinsic flexors of the thumb and fingers creates a grasp pattern between the thumb and fingers, resulting in a lateral pinch.^4^

**Figure 1:**
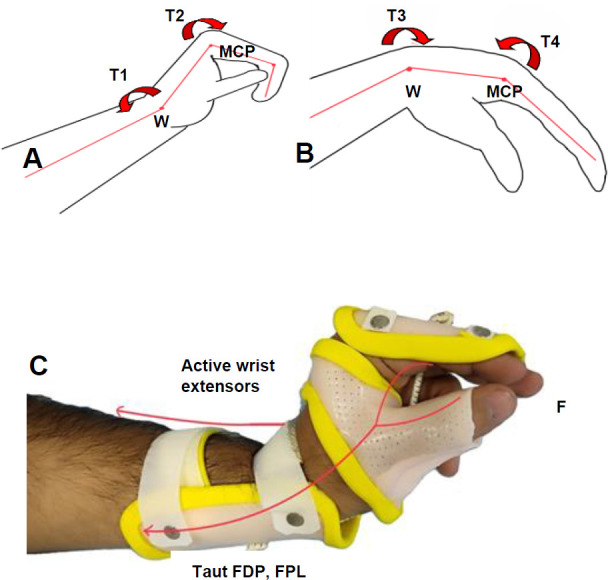
Biomechanics of a tenodesis WHO. **A**: Flexion of the fingers with the extensor torque at the wrist; **B**: Extension of the fingers with the flexor torque at the wrist; **C**: Passive stretch of the finger flexors with active wrist extension. **F**: Three-jaw chuck force; **FDP**: Flexor digitorum profundus; **FPL**: Flexor pollicis longus; **MCP**: Metacarpophalangeal joint; **T1**: Wrist extension torque; **T2**: Metacarpophalangeal joint flexion torque; **T3**: Flexion torque at the wrist; **T4**: Extension torque at the MCP joint; **W**: Wrist joint.

Active flexion of the wrist causes passive extension of the fingers. As the flexor digitorum profundus and superficialis are polyarticular muscles, complete extension of the wrist leads to about 20 degrees of flexion at the distal inter-phalangeal (DIP) joints, 50 degrees of flexion at the proximal inter-phalangeal (PIP) joints and 35 degrees of flexion at the metacarpophalangeal (MCP) joints in healthy individuals. Gravity can be used to flex the wrist, which stretches the finger and thumb extensors and thus causes the hand to open. When the wrist is actively extended, passive stretch in the flexor digitorum profundus (FDP) and superficialis and flexor pollicis longus (FPL) effectively flexes the finger and thumb.^5^ The wrist extension torque (T1) produced by active wrist extension, leads to the development of a counter-clockwise torque at the MCP joints (T2). This torque at the MCP joints is balanced with the three-jaw chuck force (F) at the static pinch between the first 3 digits (**Figure 1**).^6^

However, disruptions in tenodesis often result from altered muscle balance due to neurological deficits. This can lead to an inability to grasp objects effectively, diminishing a patient's autonomy and independence. To address this issue, orthoses have been explored to facilitate tenodesis grasp and improve hand function. The strength of the grasp depends on the amount of wrist extensor torque. A tenodesis wrist hand orthosis utilizes the natural tenodesis grasp as well as enhances this grasp by pulling the fingers towards the thumb using a connecting lever arm.^7^

The tenodesis wrist hand orthosis is a dynamic WHO that aims to enhance hand function by utilizing the natural mechanical advantage provided by intact wrist extensor muscles. The conventional tenodesis orthosis, also known as the flexor-hinge orthosis (FHO), was made of metal. The plastic tenodesis WHO developed at Rehabilitation Institute of Chicago (RIC) was a modification of traditional high temperature thermoplastic WHO developed in 1960.^7^

While metals have been used in orthoses for many decades, the use of plastics in orthoses has now become more prevalent. Plastics can be divided into two categories: thermoplastic and thermosetting. The modified tenodesis wrist hand orthosis used in this study is made of low-temperature thermoplastic that becomes malleable at a temperature of less than 149 °C. Unlike the high temperature thermoplastic RIC tenodesis WHO, a negative cast is not required in fabricating this orthosis.^8,9^ Thus, the fabrication of this modified tenodesis WHO requires less time and can be easily remoulded.

Traditional tenodesis WHOs have shown promising benefits in helping people with SCI to perform grasp and release movements. However, modifications to the design and materials of the orthosis could enhance its effectiveness. These adjustments should consider factors such as comfort, flexibility, affordability, and the specific needs of each patient. Some variations include ratchet-driven FHOs,^10^ carbon dioxide-powered FHOs,^11^ electric motor-driven FHOs, and cable-operated FHOs.^12^ Some modifications of FHO for individuals with higher-level SCI who lack voluntary wrist extension and have poor hand function include the ratchet FHO, McKibben FHO, electric motor-driven FHO, and shoulder harness-driven FHO.^6^

Recent advancements in WHOs for patients with tetraplegia have led to improved functionality and usability. The Sequential Advancing Flexion Retention Attachment (SAFRA) was developed for patients with tetraplegia with a muscle power of 2/5 or 3/5 in the wrist extensors. The SAFRA was integrated into a standard wrist-driven flexor hinge WHO with an adjustable tenodesis bar, eliminating the need for externally powered devices to achieve prehension.^13^ In South Korea, a novel 3D-printed hand orthosis controlled by electromyography (EMG) signals was tested on 10 patients, showing improvements in hand function.^14^ Wearable robotic hand exoskeletons and gloves have also been developed for patients with tetraplegia.^15,16^ However, most of these modified WHOs were bulky and patients encountered issues with donning them.

In the Indian setting, there is a need for a more affordable and comfortable alternative to the tenodesis WHO that should be easy to manufacture. The use of thermoplastic materials that become pliable when heated can expedite the production of the orthosis, thereby reducing overall costs. We developed a modified form of tenodesis WHO in response to these demands. This observational study was aimed at determining how this alteration affects manual ability among people suffering from SCI.

## METHODOLOGY

A prospective, single-arm observational study was conducted at a tertiary care center in central India to determine the effectiveness of a modified tenodesis wrist-hand orthosis (WHO) and assess patient satisfaction among individuals with spinal cord injury (SCI). All patients with SCI admitted to the Physical Medicine and Rehabilitation department were assessed, and those meeting the inclusion criteria (**Table 1**) were enrolled in the study through convenience sampling. A total of 35 individuals with SCI were screened by the primary investigator, of whom 22 patients were enrolled in the study. The study was approved by the institutional ethics committee (Institutional Human Ethics Committee, AIIMS Bhopal), and written informed consent was obtained from all participants before enrolment.

**Table 1:** Subject inclusion and exclusion criteria.

INCLUSION CRITERIA	EXCLUSION CRITERIA
1) Spinal cord injury with wrist extensor power of at least 3/5^*^ 2) Non-prehensile^†^ 3) Flexible wrist, metacarpophalangeal, and interphalangeal joints^‡^	1) Impaired cognitive function 2) Recent fracture 3) Complex regional pain syndrome 4) Neuropathy affecting the upper limb (ulnar/median/radial neuropathy or brachial plexopathy) 5) Arthritis in the wrist and joints of the fingers 6) Acute burn injury to the wrist and hand 7) Grade 2, 3, or 4 spasticity in the wrist or finger flexors or extensors¶

* Power was graded based on the Medical Research Council^17^;

† Non-prehensile was defined as the inability to seize or grasp an object, based on Napier’s^18^ classification;

‡ Full passive range of motion at the wrist with 70 degrees of extension and 80 degrees of flexion and at least 20 degrees of flexion at the DIP, 50 degrees of flexion at the PIP, and 35 degrees of flexion at the MCP joints;

¶ spasticity was assessed by the modified Ashworth scale.

The neurological level or completeness of the injury was not used as a criterion for inclusion or exclusion. The tenodesis action utilized by this modified tenodesis WHO requires active wrist extension. Therefore, individuals with at least grade 3/5 strength in their wrist extensors irrespective of injury level or completeness, were considered potential candidates.

Each extremity of subjects was screened separately. If both sides lacked prehensile ability and had a power of 3/5 or more in wrist extensors, bilateral modified tenodesis WHO was prescribed.

For all patients, the modified tenodesis WHO was fabricated and fitted by an orthotist and prosthetist with 14 years of experience.

A custom molded, comfortable, lightweight modified tenodesis WHO was designed using 2 mm low-temperature thermoplastic at a tertiary care rehabilitation center in central India, employing tenodesis grasp and biomechanical principles.

**Figure 2:**
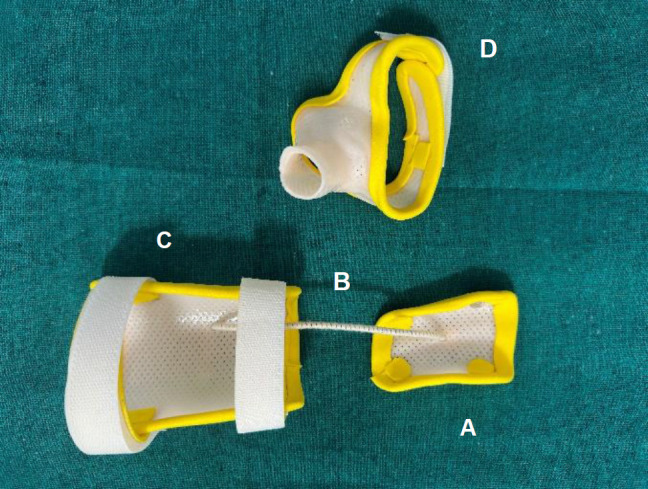
Modified tenodesis wrist hand orthosis. The orthosis is made of low-temperature thermoplastic material and consists of four components. **A**: Dorsal finger plate for digits 2 and 3; **B**: An elastic lacer that serves as the wrist component and facilitates the translation of tenodesis; **C**: Distal palmar forearm component; **D**: Short opponens component.

As illustrated in **Figure 2**, the modified tenodesis WHO is comprised of four components: segment (A) encompasses the middle and distal interphalangeal joints of the index and middle fingers; segment (B) is an elastic lacer that anchors to the finger and forearm components, and facilitates translation of tenodesis; segment (C) encompasses the palmer wrist and forearm; and segment (D) a short opponens component, maintains the proximal palmer arc and aligns the thumb in opposition.

Velcro^®^ straps were used to secure the distal palmar forearm component, dorsal finger plate, and short opponens component, allowing adjustments for patient comfort. An elastic lacer, 2 mm thick, connected the volar surface of the dorsal finger plate to the volar surface of the distal palmar forearm component. The inclusion of the elastic lacer eliminated the need for actuating rod and artificial joint components as opposed to the conventional flexor hinge WHO, thus reducing the complexity of the orthosis.^6^

The weight of this WHO was approximately 80 grams (75–85 grams), slightly lighter than the conventional WHO (approximately 113 grams) and heavier than the RIC tenodesis WHO (42.5 grams).^6^ The orthosis was customized according to each individual’s shape and size, with a weight difference of 5–10 gram corresponding to the size variation. The cost of the orthosis is $4.50 USD, inclusive of material costs and labor charges, making it more economical than the RIC tenodesis WHO, priced at around $39.95 USD.^19^

After applying the modified tenodesis WHO, participants were trained for 2 weeks regarding the proper usage of the orthosis, and specific task-oriented training was given to the patients. All participants required the help of the caregiver for donning and doffing. During this period, the accompanying family members/caregivers were also taught proper donning and doffing of the orthosis. Proper care of the orthosis, avoidance of heat, cleaning with cold water, the need for the orthosis and the mechanism of use of the orthosis were explained to the individual. The participants were allowed to ask their queries regarding the orthosis throughout the sessions.

**Figure 3:**
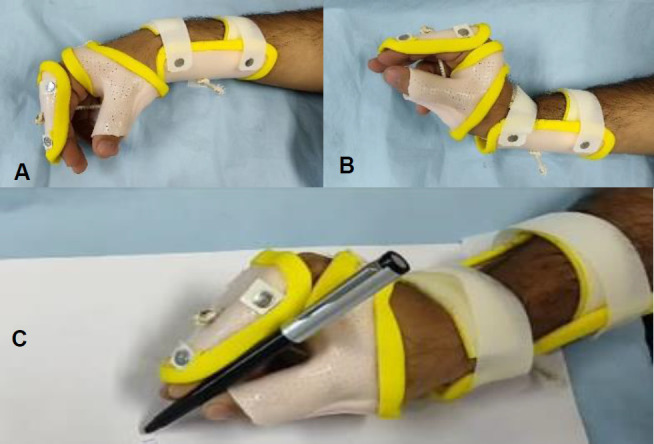
A patient with tetraplegia demonstrating use of the modified tenodesis WHO. **A**: Wrist flexion; **B**: Wrist extension and three-jaw chuck grasp; **C**: Holding a pen.

All the study participants received intensive training for 45 minutes per day for 2 weeks under a trained Occupational Therapist. The training included wrist extensor and flexor strengthening exercises, peg-socket activities, grasp and release activities (**Figure 3**), and activities of daily living (ADL) training in a simulated environment.

The demographic and clinical data of the participants were documented. The outcome measures included the Duruöz Hand Index (DHI) and the Orthotics and Prosthetics User’s Survey (OPUS) satisfaction with device and services. The DHI is a valid tool for patients with tetraplegia, and it is used to assess hand function.^20^ The DHI is a Likert scale that comprises 18 questions pertaining to hand activities that can be assessed based on the patient’s perspective. The OPUS satisfaction with device and services subjectively measured satisfaction with the device and the services using a 21-item survey.^21^

While assessing DHI, a few questions pertained to the use of a single hand, such as picking up a coin from the tabletop. These activities were assessed on the side on which the orthosis was prescribed. In those patients who were fitted with bilateral modified tenodesis WHO, the dominant hand was assessed for activities requiring a unilateral hand. The dominant hand was determined based on pre-injury hand dominance. Outcome measures were recorded without the orthosis initially (week 0) and with the orthosis at 6 weeks and 12 weeks by the same treating physician at each follow-up.

### Statistical analysis

Categorical variables such as gender, neurological level of injury (NLI), and the side of use for the modified tenodesis WHO were presented as counts and percentages. Continuous variables, including age and duration since injury, were expressed as means, standard deviations, and ranges. Mean values were taken for individual items of the OPUS satisfaction with device and services, while a median value was taken for the total OPUS satisfaction with device and services score to represent the central tendency, as it is a Likert type of scale. A Wilcoxon signed-rank test was employed to assess the change in mean DHI scores of all 22 patients at 6 weeks and 12 weeks compared to baseline. Statistical significance was set at p < 0.05 and analysis was conducted using SPSS version 21.0.

## RESULTS

Twenty-two individuals with tetraplegia were included in the study, of whom 18 were male and 4 were female. Eleven subjects were fit with the modified tenodesis WHO on the right upper extremity, whereas 6 subjects were fit with the device on the left upper extremity and 5 were fit with the device on both upper extremities (**Table 2**).

**Table 2:** Subjects demographic data.

Item	Number	Percentage (%)
**Gender**		
Male	18	81.8
Female	4	18.2
^*^NLI: Neurological level of injury		
C2	1	4.5
C4	3	13.7
C5	11	50.0
C6	5	22.7
C7	2	9.0
**Modified tenodesis WHO side**		
Right	11	50.0
Left	6	27.3
Bilateral	5	22.7
Dominant	12	54.6
Non-dominant	5	22.7
Dominant and non-dominant	5	22.7
Mean age (range)	36.7 (20–64) years	
Mean duration since injury (range)	9.8 (3–34) months	

**Figure 4:**
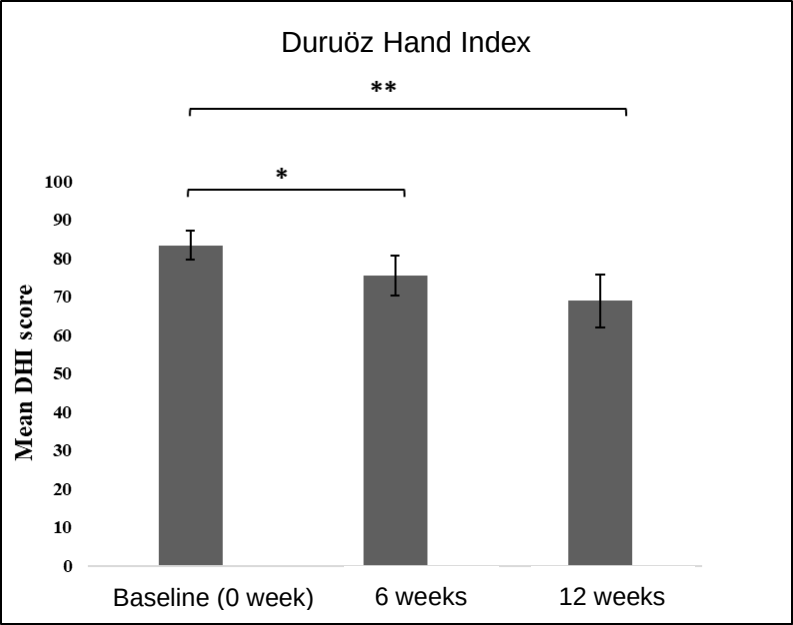
Duruoz Hand Index (DHI). ^*^p<0.001 for difference in mean DHI scores at 0 and 6 weeks; ^**^p<0.001 for difference in mean DHI scores at 0 and 12 weeks. The total DHI score (out of 90) was calculated for all 18 items, while each item was scored from 0*5 (0: Yes, without difficulty; 1: Yes, with a little difficulty; 3: Yes, with much difficulty; 4: Nearly impossible to do; 5: Impossible).

The DHI score at admission was collected as the baseline. The mean ± standard deviation for DHI at baseline (no orthosis) was 83.4 ± 3.72, while it was 75.5 ± 5.12 at 6 weeks and 68.9 ± 6.92 at 12 weeks with the modified tenodesis WHO, respectively. Compared to the baseline, there was a significant improvement in DHI at 6 weeks (p<0.001) and 12 weeks (p<0.001) with the use of the modified tenodesis WHO (**Figure 4**).

Among all the items on the DHI scale, the activity performed with the least difficulty while using the modified tenodesis WHO was pricking items with a fork. Significant improvement (**Table 3**) was perceived in performing all functional activities of the DHI scale using the modified tenodesis WHO at 12 weeks as compared to baseline except unscrewing the lid of a jar and peeling a fruit. The most significant functional improvement at 6 weeks compared to baseline was observed in buttoning a shirt (p=0.000), whereas the greatest enhancement was noted in turning a key in a lock at 12 weeks (p=0.000) compared to baseline (**Table 3**).

**Table 3:** Mean and standard deviation (SD) of each DHI subitem at 6 weeks and 12 weeks as compared to the baseline.

	DHI mean ± SD at 0 week	DHI mean ± SD at 6 weeks	DHI mean ± SD at12 weeks	p^*^	p^**^
Hold a bowl	4.72 ± 0.63	4.64 ± 0.66	4.23 ± 0.92	0.157	0.009
Seize a full bottle and raise it	4.90 ± 0.29	4.77 ± 0.43	4.32 ± 0.72	0.083	0.004
Hold a plate full of food	4.72 ± 0.55	4.59 ± 0.59	4.14 ± 0.89	0.083	0.006
Pour liquid from a bottle into a glass	4.86 ± 0.35	4.68 ± 0.65	4.32 ± 0.78	0.102	0.006
Unscrew the lid from a jar opened before	4.72 ± 0.70	4.68 ± 0.72	4.59 ± 0.80	0.317	0.180
Cut meat with a knife	4.40 ± 0.91	3.55 ± 0.60	3.41 ± 0.67	0.000	0.000
Prick things well with a fork	3.77 ± 0.81	2.73 ± 0.46	2.45 ± 0.51	0.002	0.000
Peel fruit	4.90 ± 0.29	4.91 ± 0.29	4.68 ± 0.57	1.000	0.059
Button your shirt	4.50 ± 0.86	3.32 ± 0.89	3.05 ± 0.95	0.000	0.000
Open and close a zipper	4.27 ± 0.83	3.86 ± 0.94	3.59 ± 0.67	0.003	0.000
Squeeze a new tube of toothpaste	4.50 ± 0.86	3.95 ± 0.90	3.64 ± 0.66	0.006	0.000
Hold a toothbrush efficiently	4.72 ± 0.46	4.36 ± 0.79	3.32 ± 0.84	0.023	0.000
Write a short sentence with a pencil or ordinary pen	4.54 ± 0.67	3.86 ± 0.83	3.27 ± 0.83	0.004	0.000
Write a letter with a pencil or ordinary pen	4.90 ± 0.29	4.32 ± 0.84	3.77 ± 0.81	0.009	0.000
Turn around doorknob	4.72 ± 0.63	4.68 ± 0.72	4.41 ± 0.80	0.317	0.020
Cut a piece of paper with scissors	4.90 ± 0.43	4.86 ± 0.47	4.73 ± 0.55	0.317	0.046
Pick up coins from a tabletop	4.31 ± 0.84	3.95 ± 0.95	3.59 ± 0.73	0.011	0.000
Turn a key in a lock	4.90 ± 0.29	3.82 ± 0.59	3.36 ± 0.49	0.000	0.000

p^*^ = p-value between means at 0 and 6 weeks.

p^**^= p value between means at 0 and 12 weeks. The p-values are calculated using the Wilcoxon signed-rank test.

**Figure 5:**
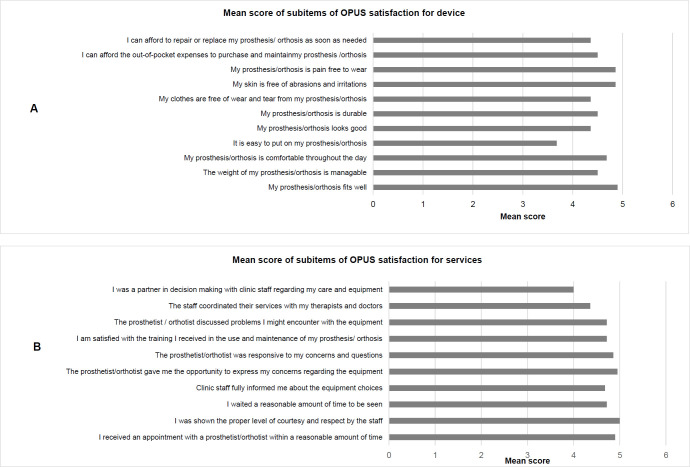
OPUS satisfaction scores. **A**: Satisfaction for device; **B**: Satisfaction for services. The response to each item was scored as follows: 5= Strongly agree; 4= Agree; 3= Neither; agree nor disagree; 2= Disagree; 1= Strongly disagree.

The median for the OPUS device satisfaction score was 50, pertaining to a Rasch score of 68.8 and the median for the OPUS satisfaction for services score was 46, with a Rasch score of 72.7. Among the subitems of the OPUS device satisfaction, the maximum satisfaction was experienced with respect to the fit of the orthosis, while the least satisfaction was experienced with donning the orthosis (**Figure 5**A)

All patients expressed the highest satisfaction with the behavior of staff (i.e., courteous and respectful) and expressed the lowest satisfaction with their own decision-making process regarding the orthosis (**Figure 5**B).

## DISCUSSION

The results of this prospective observational study demonstrated significant improvements in hand function among patients with SCI who utilized the modified tenodesis WHO.

The effectiveness of this modified tenodesis WHO in enhancing hand function is demonstrated by a significant improvement in the DHI scores after 6 and 12 weeks compared to baseline. The notable enhancements in activities such as opening a lock and buttoning a shirt underscore the importance of the modified tenodesis orthosis in improving pinch grip. This orthosis enables skillful placement of objects between the thumb and fingers, facilitating the manipulation of small items like keys, forks, buttons, and pens, ultimately enhancing overall hand function. However, the omission of the digits 4 and 5 from the modified WHO may have contributed to limitations in spherical and cylindrical grasp as these types of grasp patterns typically require engagement of all five digits. Thus, there were limited gains in activities such as unscrewing a jar, holding a bowl, or lifting a bottle. Hence, a limitation of the orthosis is that it restricts grasping that requires all five digits, likely necessitating the user to employ alternative movements to compensate for this limitation. Similar results with improvements in hand function using various designs of a tenodesis WHO have been previously seen. In a study by Meyer et al., the application of a flexor hinge WHO enabled a 43-year-old teacher with a complete C6-level SCI to return to work and perform domestic activities, such as cooking.^22^ In the same study, another woman who had a C6-level complete lesion was able to return to the farm and perform activities like cooking, sewing, and knitting with bilateral flexor-hinge WHO. A modified wrist-driven flexor hinge WHO with a dial lock, allowing the orthosis to function at different ranges of motion at the wrist joint, was studied by Rout et al.^23^ They observed that without the orthosis, participants were able to lift 50 grams of weight, while with the orthosis, there was an increase in the ability to lift the weight up to 300 grams. In our study, significant improvement in the ability to raise a full bottle and plate full of food indicates an increased ability to lift weight, but the improvement in such activities at 6 weeks was not significant.

In a study by Yoo et al. a 3D-printed myoelectric wrist hand orthosis was assessed for improvement in hand function of persons with SCI,^14^ representing one of the latest advancements for patients with tetraplegia. Their findings also demonstrated improvement in eating with the 3D-printed orthosis, although there were no significant gains in dressing or grooming abilities.

Batra et al. compared the efficacy of biofeedback to use of a tenodesis WHO in persons with C5-C6 SCI.^24^ A significant improvement in the ability to lift weights and in the Functional Independence Measure (FIM) was achieved in both the groups, but biofeedback was found to be more effective. Based on these promising findings, biofeedback could potentially be used in conjunction with the use of the modified tenodesis WHO in future studies to investigate its efficacy in facilitating clinical outcomes.

Assessment using the OPUS satisfaction with device and services in our study revealed that participants expressed high satisfaction levels with the courteous behavior of staff, prompt appointment scheduling, and appropriate fit of the orthosis. The thermoplastic material's easy moldability directly over the skin ensured an appropriate fit, and patients reported satisfaction with training and ease of use. However, a notable area of dissatisfaction was experienced during the donning and doffing of the orthosis. Despite having fewer components compared to traditional flexor hinge orthosis, patients with impaired hand function often require assistance from caregivers due to weakness in both upper extremities, indicating a need for further modifications to improve independent donning and doffing capabilities.

The results of this study hold significant clinical implications for patients with SCI undergoing hand function rehabilitation, potentially enhancing their participation in both personal and professional activities.

Furthermore, investigating the long-term effects of the modified tenodesis WHO on hand function and exploring its applicability across various levels and severities of SCI are avenues for further research. Additionally, incorporating qualitative measures to capture participants’ perceptions and experiences with the WHO could provide valuable insights into its acceptability and usability.

### Limitations

Several limitations warrant consideration. The lack of a control group precludes definitive causal conclusions regarding the efficacy of this wrist hand orthosis. Additionally, the relatively short intervention period may have influenced the magnitude of the observed improvements. Future studies could mitigate these limitations by incorporating randomized controlled designs and extended intervention durations.

## CONCLUSION

In conclusion, the results of this observational study provided evidence supporting the potential efficacy of a modified tenodesis WHO in improving hand function among patients with SCI, particularly in activities such as eating with a fork, buttoning a shirt, locking with a key, holding a toothbrush, and writing with a pen. The observed enhancements in functional independence suggest that this intervention could significantly contribute to hand rehabilitation strategies for individuals with SCI. The participants had maximum satisfaction with the behavior of the staff, prompt appointments and appropriate fit of the device, while they were most concerned with donning difficulty. The findings encourage further investigation and application of the modified tenodesis WHO in clinical practice.

## DECLARATION OF CONFLICTING INTERESTS

The authors disclose that they have no personal or financial ties to any companies or people that could have impacted their research.

## AUTHORS CONTRIBUTION

- **Swapnil P. Sonune:** Conceptualization, Original drafting, Writing, Data collection, Statistical analysis.
- **Anyesha Saha:** Writing, Data collection, Statistical analysis, Revising the manuscript.
- **Joshi Niravkumar Ganpatram:** Writing, Data collection, Revising the manuscript.
- **Smita Pathak:** Designing orthosis.
- **Prasenjit Bhadra:** Writing, Data collection, Revising the manuscript.
- **Gaurav Goel:** Writing, Data collection, Revising the manuscript.

All authors reviewed the manuscript and approved the final version.

## SOURCES OF SUPPORT

There was no funding for this research.
